# Application of Triboelectric Nanogenerator in Fluid Dynamics Sensing: Past and Future

**DOI:** 10.3390/nano12193261

**Published:** 2022-09-20

**Authors:** Leo N. Y. Cao, Zijie Xu, Zhong Lin Wang

**Affiliations:** 1 CAS Center for Excellence in Nanoscience, Beijing Key Laboratory of Micro-Nano Energy and Sensor, Beijing Institute of Nanoenergy and Nanosystems, Chinese Academy of Sciences, Beijing 101400, China; 2 School of Nanoscience and Technology, University of Chinese Academy of Sciences, Beijing 100049, China; 3 School of Materials Science and Engineering, Georgia Institute of Technology, Atlanta, GA 30332-0245, USA

**Keywords:** triboelectric nanogenerators, fluid dynamics sensing

## Abstract

The triboelectric nanogenerator (TENG) developed by Z. L. Wang’s team to harvest random mechanical energy is a promising new energy source for distributed sensing systems in the new era of the internet of things (IoT) and artificial intelligence (AI) for a smart world. TENG has many advantages that make it suitable for a wide range of applications, including energy harvesting, environmental protection, wearable electronics, robotics, and self-powered sensors. Sensing as an important part of TENG applications is gradually expanding, with the in-depth study of TENG sensing in its working principle, material selection, processing technology, system integration, surface treatment, and back-end algorithms by researchers. In industry and academia, fluid dynamics sensing for liquid and air is urgently needed but lacking. In particular, local fluid sensing is difficult and limited to traditional sensors. Fortunately, with advantages for ordinary TENGs and TENGs as fluid dynamics sensors, fluid dynamics sensing can be better realized. Therefore, the paper summarizes the up-to-date work on TENGs as fluid dynamics sensors, discusses the advantages of TENGs as fluid dynamics sensors in-depth, and, most importantly, aims to explore possible new key areas to help guide the future direction of TENG in fluid dynamics sensing by addressing the key challenges.

## 1. Introduction

With the rapid development of the Internet of Things (IoT) and artificial intelligence (AI) for a smart world, distributed sensing systems, which are the foundation of the fourth industrial revolution, are the most important developments in hardware in the era. The continuous development and prosperity of distributed sensing systems rely on distributed renewable sources of energy such as solar power, wind power, and mechanical vibration [1]. The triboelectric nanogenerator (TENG) developed by Z. L. Wang’s team to harvest random mechanical energy is a promising new energy source in the new era because triboelectric electrification is ubiquitous with a wide selection of materials [2]. Compared to externally powered sensors, the development of self-powered active sensors powered by TENG is revolutionary. In addition, TENG has many other advantages such as an abundant choice of materials, low assembly requirement, and flexibility [3], making it suitable for many application areas, including energy harvesting, environmental protection, wearable electronics, robotics, and self-powered sensors [4].

An important part of TENG applications is sensing that focuses mainly on active mechanical and chemical sensors in the early stages. Additionally, early TENG can actively detect the static and dynamic processes from mechanical agitation with potential smart skin applications [5]. With the in-depth study of TENG sensing in its working principle, material selection, processing technology, system integration, surface treatment, and back-end algorithms by researchers, the application fields of TENG sensing are gradually expanding, especially fluid dynamics sensing. Fluids, including gases and liquids, account for two of the three phases of matter and tend to flow, meeting the working principle of TENG: mechano-driven. Most importantly, fluid dynamics sensing is urgently needed in industry and academia. In particular, local fluid sensing is difficult and limited to traditional sensors.

**Figure 1:**
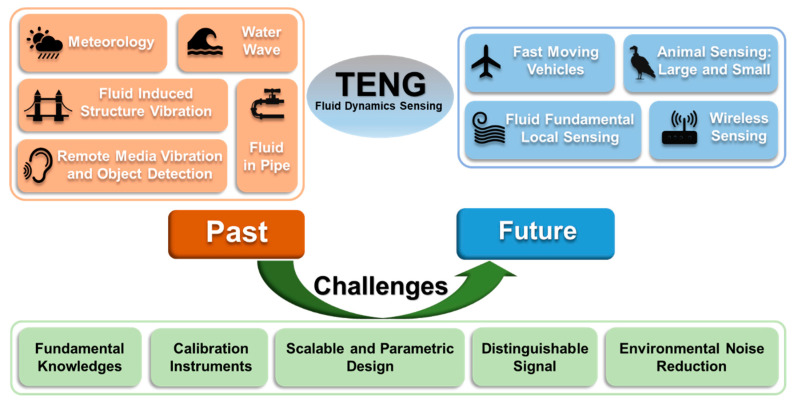
Opportunities and challenges of triboelectric nanogenerators for fluid dynamics sensing, from traditional fields to future technologies.

Fortunately, with advantages for ordinary TENGs (discussed later in this section) and TENGs as fluid dynamics sensors (introduced in Section 4), fluid dynamics sensing can be better realized. For example, the self-powered characteristics of TENG can simplify the sensor structure, so that the disturbance of the sensor for the flow field is reduced. In addition, the diversity of material choices and the sensitivity to external stimuli give TENG an advantage over other existing sensing schemes in complex fluid environment sensing. Therefore, the objective of the paper is to summarize the recent work on TENG as fluid dynamics sensors, and, most importantly, explore possible new key areas to help guide the future direction of TENG in fluid dynamics sensing by addressing the key challenges (Figure 1).

The review is structured as follows: in, the principles and advantages of TENG are first introduced (Section 2). We then summarize the up-to-date work on TENG primarily as fluid dynamics sensors for the local fluid phenomenon and environment (Section 3); in Section 4, we explore the possibility of TENG and offer guidance by addressing the needs of industry and academia by leveraging the advantages of the TENG as fluid dynamics sensors, focusing more on the local sensing of fluids. Finally, in Section 5, we discuss the challenges TENG faces to reach the milestone and become suitable, and practically applicable sensors with distinct uniqueness and advantages.

## 2. Introduction of TENG

**Figure 2:**
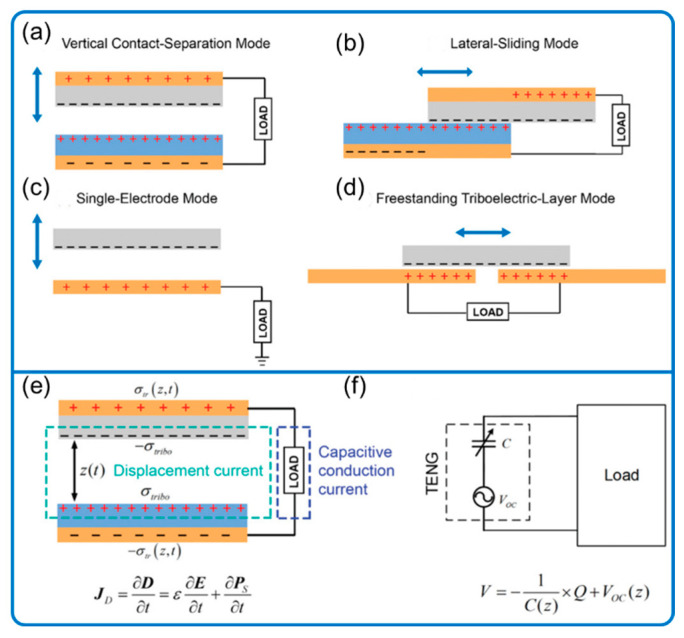
The four basic modes of TENG and its equivalent model. (**a**) Vertical contact separation mode, (**b**) lateral sliding mode, (**c**) single-electrode mode, and (**d**) freestanding triboelectric-layer mode. (**e**) The displacement current model of a contact-separation mode. (**f**) TENG’s equivalent electrical circuit model. Reproduced with permission. Copyright 2018, Wiley Online Library [1].

Coupling the triboelectric effect and electrostatic induction, TENG was first invented by Wang’s group in 2012 (Figure 2) to generate electricity through harvesting environmental mechanical energy that is ubiquitous but often wasted. More specifically, the electricity is converted by electrostatic induction through the electric field change, which is induced by mechanical separation after triboelectric or contact electrification transfers electrons from one surface of contacting materials to another according to the quantum mechanical transition model [6]. The model states that when two materials approach an atomically close distance, electrons move toward the lowest available states due to strongly overlapping electron waves. The electron transfer model could be extended from solid–solid to liquid–solid, liquid–gas, and even liquid–liquid cases [7]. 

### 2.1. Principle of TENG

The fundamental physics model of TENG was presented according to Maxwell’s displacement current in 2017 [4,8,9,10]. The displacement current is from a transient electric field and media dielectric polarization and drives the conversion of mechanical energy into electricity. Mainly due to the independence of the surface charges on the electric field, a new term *P_s_*, known as the Wang term, is added to Maxwell’s equations to explain TENG’s working mechanism [4]. The displacement current density (*J_D_*) is then expanded as follows:

$$J_{D}=\varepsilon \frac{\partial E}{\partial t}+\frac{\partial P_{S}}{\partial t}=\varepsilon_{0}\frac{\partial E}{\partial t}+\frac{\partial P}{\partial t}+\frac{\partial P_{S}}{\partial t}$$

where *E*, *P*, and *P_s_* represent the electric field, medium polarization vector, and the added term from the presence of electrostatic surface charges, respectively; *ε* and *ε_0_* are the permittivity of the dielectrics and vacuum, respectively. The first term (ε∂E/∂t) represents the electromagnetic wave, which is predicted to emit from a high-frequency TENG [4] and later detected [11]. In addition, other wireless signals created by TENG were also detected and summarized in Wang et al. [12].

TENG can be categorized into four basic modes according to their electrodes and motion patterns: vertical contact separation, lateral sliding, single-electrode, and freestanding triboelectric-layer modes (Figure 2) [13]. In general, electrons are transferred back and forth from one electrode to another (generating electrical current) due to the change of electrical field caused by the mechano-driven location change of triboelectric materials. The electron transfer is slightly different for the single-electrode mode (Figure 2c) since it has only one electrode: the only electrode exchanges electrons with the ground. Each mode has its advantage in energy harvesting, manufacturing, and robustness [3,13]. In addition, the various motion patterns give more flexibility to the sensors’ working mechanism, design, and manufacturing. For example, the single-electrode mode for the TENG as a sensor may give the lowest energy output and thus signal-to-noise ratio (SNR) but can sense the object directly with the easiest setup since the triboelectric material, possibly the object being sensed (e.g., liquid), does not necessarily belong to the system. The displacement current model of contact separation mode and the equivalent electrical circuit model is described in Figure 2e,f, respectively.

### 2.2. Advantages of Ordinary TENG

In addition to multiple modes of operation, ordinary TENG has many other advantages, including wide material availability, lightweight, low cost, and high efficiency even at low operating frequencies. In addition, its sensitivity to external incentives and self-powered characteristics also broaden its scope of application. Advantages for TENGs as fluid dynamics sensors will be introduced in Section 4.

In principle, any material with different charge affinities can be used to construct TENG. Thus, due to the wide material selections, we can conveniently adjust the basic mechanical, physicochemical, and biological properties of the TENG to suit various application situations. Furthermore, TENGs are reported to generate power densities as high as several hundred Wm^−2^ which is sufficient to drive many small electronics, allowing self-powered sensing networks. In addition, thanks to the recent development, wireless data induced by TENG can be received in many ways (discussed in Section 4), providing an alternative framework for sensor network construction and data transmission [12].

## 3. TENG for Fluid Dynamics Sensing: Past

In physics and engineering, fluid dynamics is a branch of fluid mechanics that describes the flow of fluids, including aerodynamics (study of gases in motion) and hydrodynamics (study of liquids in motion). Here, we divide the fluid dynamics parameters that may be sensed by TENG sensors into two groups by scale: large- and local-scale properties. The large-scale properties include ambient fluid motions, such as wind, rain, and water wave, and their speed, direction, and pressure; the local-scale properties include the flow pattern and force under various situations on the local scale, such as laminar and turbulence flow and their development, boundary layer and its separation, flow pattern around immersed bodies, rotational fluid such as vortex, and streamlines in the local flow field (Figure 1).

This section summarizes the recent studies using TENG sensors to sense the abovementioned fluid dynamics parameters. According to the application scenarios, we divided the fluid dynamics TENG sensors into five categories: 1 meteorology parameters, 2 fluids in pipes and tunnels, 3 remote media vibration and moving object, 4 sea wave motion, and 5 structure vibration due to moving fluid. As seen in the classification, a large part of the current work in fluid dynamics sensing involves large-scale properties. Note that this review only covers research where TENG is or could be used as a sensor or even battery-less sensor and excludes studies that merely use TENG as a power source to run commercial sensors. In addition, a few reviews summarized some overlapping or related contents to this article, but with different focuses: Gao et al. summarized sensors of wind and water in pipes in their review of triboelectric mechanical sensors [3]; Tang et al. discussed liquid and droplet content sensors using TENG [14]; Nguyen et al. summarized fluid-based TENG as power sources [15].

### 3.1. Meteorology-Related Sensing

Targeting on developing alternative smart tools for conventional weather reporting and monitoring (e.g., wind speed and direction, and rainfall amount), researchers have worked on meteorology-related TENG sensors (mostly battery-less sensors in the subsection) based on traditional measurement methods.

(a) Wind speed and direction: conventional wind cups and turbine.

**Figure 3:**
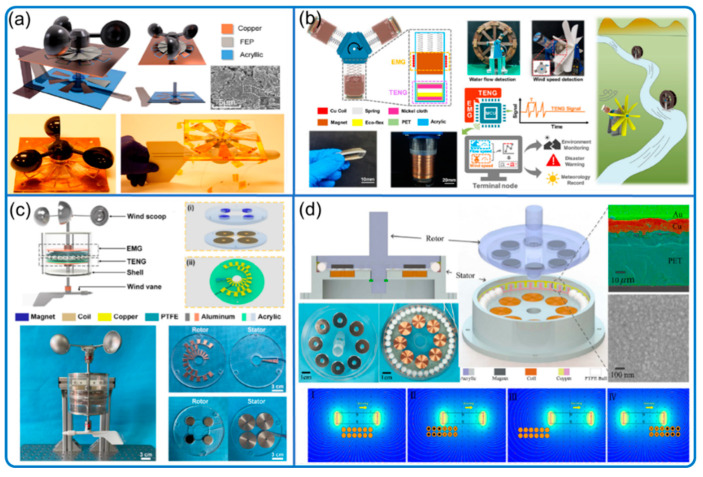
Sensing of wind speed with direction: conventional wind cups and turbine. (**a**) Soft friction system with wind vane. Reproduced with permission. Copyright 2018, American Chemical Society [18]. (**b**) Unmanned environment monitoring system. Reproduced with permission. Copyright 2021, Elsevier [21]. (**c**) Wind direction sensing with Gray code. Reproduced with permission. Copyright 2021, American Chemical Society [19]. (**d**) Rolling mode sensor with electromagnetic generator (EMG) direction indicator. Reproduced with permission. Copyright 2020, Elsevier [20].

One of the TENG sensor types for wind speed and direction measurement is similar to the wind vector sensor system (wind vane and cups or turbine) commonly used in traditional weather forecasting, where the rotation speed of the wind cups or turbine correlates well with wind speed (Figure 3). More specifically, they employed wind cups or wind turbines as the motion generator, rotational TENGs with graded electrodes for wind speed information, and an optional wind vane indicator providing possibly high-resolution wind direction.

Since the wind speed correlates well with the wind cup rotational speed (rpm), which corresponds to the TENG signal frequency, many of the rotational sensors use TENG signal frequency to correlate with and represent wind speed. Using signal frequency or peak counts to record data, similar to the encoding techniques in telecommunication, is also advantageous for noise reduction and data fidelity [16,17] compared to signal amplitude, which can be affected by environmental factors such as humidity and temperature.

To indicate constantly changed wind direction, they normally use wind vane, together with coded electrodes or other signal generators [18,19,20]. To differentiate multiple directions, graded signals with different levels are needed, requiring multiple signal channels in general. For example, eight independent signal channels were used to indicate eight wind directions in Wang et al. (Figure 3a) [18] and Han et al. (Figure 3d) [20]. Since the increasing number of signal acquisition channels will gradually increase system difficulty and complexity, signal encoding methods are often applied to address the problem [16,17]. For example, Zhang et al. achieved 8 wind direction detection within 2 s using only three electrical channels by encoding the electrodes with Gray code (Figure 3c), increasing the applicability and practicality drastically [19]. In addition to the wind vane method to indicate wind direction, the array of wind-direction-sensitive sensors is used and calibrated to indicate rough (low resolution) wind direction (Figure 3b) [21].

**Figure 4:**
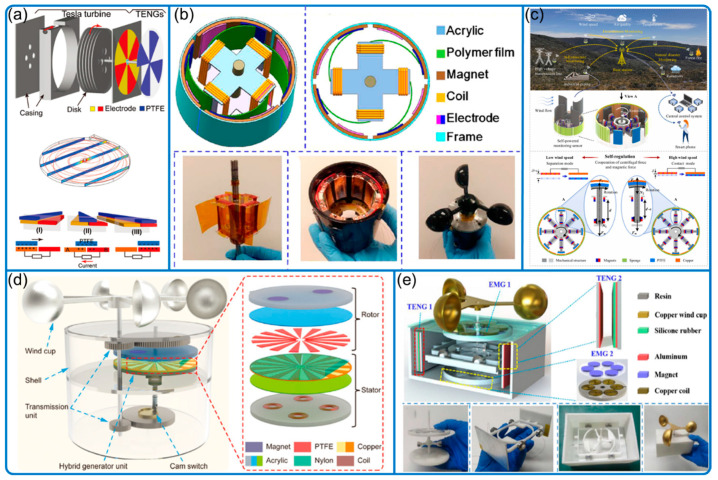
Conventional wind speed sensing with performance enhancement features. (**a**) Bladeless turbine structure. Reproduced with permission. Copyright 2018, Wiley Online Library [22]. (**b**) Ultra-low friction system. Reproduced with permission. Copyright 2018, American Chemical Society [24]. (**c**) Wind-speed-dependent self-regulation strategy. Reproduced with permission. Copyright 2022, Elsevier [26]. (**d**) Travel-controlled approach for high durability. Reproduced with permission. Copyright 2022, Wiley Online Library [25]. (**e**) Rotational to linear motion conversion. Reproduced with permission. Copyright 2020, Elsevier [23].

The detailed information of wind cup sensors is summarized in Table 1, including wind speed range, wind direction, and features. The general tested wind speed is from 2.0 to 15.0 m/s with some special cases, for example, Chen et al. (Figure 4a) developed a bladeless-turbine-based (tesla turbine) high-speed TENG sensor, up to 7500 rpm (11.0 to 28.0 L/min) [22]. Note that the upper limit of tested wind speed may be limited by the testing scenarios, methods, and apparatus; therefore, the stated wind speed may also be either inaccurate or biased if the testing apparatus and method are not well controlled. For example, only a few studies conducted standard measurements in well-controlled wind tunnels, so the wind speed value, especially the lowest starting wind speed is not claimed strictly, leaving an area to improve for sensor calibration.

Many wind speed sensors applied the freestanding mode for the TENG for better energy harvesting, while others used contact separation modes by converting rotational to linear motion [21,23], reducing device friction, wear, and minimum starting wind speed (Figure 4). Other attempts have been also tried to enhance device robustness and efficiency by applying, for example, soft contact (Figure 4b) [24], and automatic switch (Figure 4c,d) for contact and separation [25,26].

Most of the abovementioned devices can either work as a sensor themselves or harvest environmental energy to serve as small power sources for commercial sensors with low power consumption, often hybridized with electromagnetic generators (EMG) to increase power generation [18,19,25]. However, harvesting energy and sensing simultaneously in a single TENG device is difficult without any thoughtful treatment. To solve the problem, Lu et al. proposed a method of decoupling and extracting signals and energy so that the disk-type TENG can sense the wind speed, harvest wind energy, and process and send the signal to a wireless signal receiver simultaneously, achieving a truly closed-loop self-powered environmental sensing system [27].

**Table 1:** Summary of traditional wind cup and vane style TENG sensors.

Reference	Wind Speed (m/s)	Wind Direction	Features
Wang et al. [24]	3.5 to 9.0	N/A	EMG hybrid device with ultra-low friction
Chen et al. [22]	N/A	N/A	A freestanding bladeless-turbine-based (tesla turbine)
Wang et al. [18]	2.7 to 8.0	Yes	Freestanding (soft contact) disk-like
Han et al. [20]	6.0 to 12.0	Yes, EMG indicator	Ball bearing-like with graded electrodes
Fan et al. [23]	4.0 to 15.0	N/A	A cam structure to convert rotational to linear motion
Lu et al. [27]	6.0 to 12.0	N/A	Freestanding mode disk-like
Zhang et al. [19]	3.0 to 15.0	Yes, with only 3 signal channels	Coded electrodes for wind direction within 2 s
Ye et al. [28]	1.55 to 15.0	N/A	Combined 3 TENGs (flag, disk-brush soft contact, and EMG)
Zhang et al. [21]	3.0 to 15.0	Yes, array of devices	Contact separation mode in rotational device
Ma et al. [29]	2.9 to 9.1	N/A	Wheat-straw based TENG
Zou et al. [26]	2.0 to 12.0	N/A	Self-regulated contact-separation mode; can start with low wind speed
He et al. [30]	3.77 to 11.91	N/A	Disk-brush-like freestanding mode
Luo et al. [25]	3.0 to 15.0	N/A	Travel-controlled approach with cam switch

(b) Wind speed and direction: flutter- and flag-type

**Figure 5:**
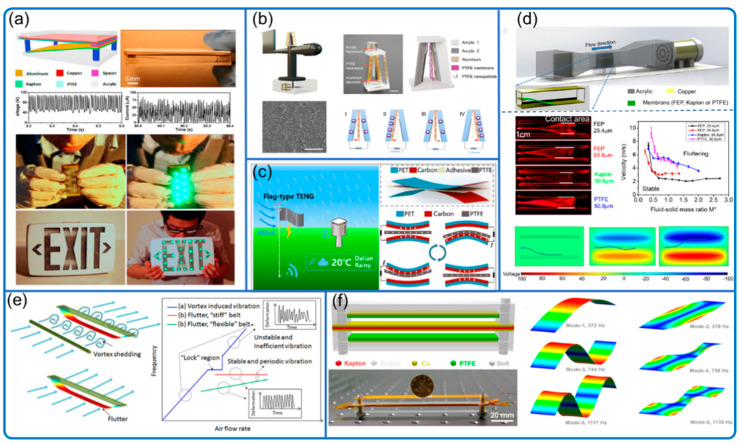
Typical flutter sensor and its mechanism discussion. (**a**) Flutter-type sensor system. Reproduced with permission. Copyright 2013, American Chemical Society [31]. (**b**) Wind vector detecting system. Reproduced with permission. Copyright 2021, Elsevier [43]. (**c**) Flag-type sensor. Reproduced with permission. Copyright 2020, Elsevier [44]. (**d**) Characterization of fluttering behavior and COMSOL simulation. Reproduced with permission. Copyright 2017, Elsevier [35]. (**e**) Theoretical model for flutter phenomenon. Reproduced with permission. Copyright 2016, AAAS [34]. (**f**) Double-clamped aero-elastic system. Reproduced with permission. Copyright 2015, American Chemical Society [39].

This part includes flutter- and flag-type sensors that are susceptible to flow-induced vibration. The devices often consist of a rectangular cross-section tunnel, two electrically connected electrodes on both sides, and a long flexible membrane (in the middle of the flow along the flow direction) that can flutter along the flow so that wind energy can be harvested and the flow information can be also sensed by the periodic electrical signals from the current due to electrostatic induction between the film and electrodes (Figure 5) [31].

The experimental phenomenon and results are solid and obvious, but the theoretical fluttering explanation is often flawed. Many researchers investigated the problem from the perspective of aerodynamics [32,33,34,35,36,37]. However, even in pure aerodynamics, the answer is not clear. There are two main mechanisms to explain the flutter effect: (1) vortex-induced vibration by adding a bluff body before the membrane and governed by the Strouhal number and (2) without a bluff body, the membrane will also flutter due to the aerodynamic flutter effect. To thoroughly investigate the fluttering effect, Bae et al. divided the fluttering status into single, dual, and chaotic modes, which are indicated by two proposed dimensionless parameters: the nondimensional velocity and dimensionless mass of the flag. These dimensionless parameters are universal and extremely useful for future researchers. In addition, a mathematical relationship between the proposed parameters and fluttering degree, e.g., the fluttering frequency, can be further developed. Chen et al. (Figure 5e) developed a unified theoretical framework for the flutter phenomenon of both stiff and flexible piezoelectric materials, and validated by a micro wind belt system analyzer, in which the airflow rate is from 64 to around 260 m/s and the vibration frequency is up to several thousand Hz [34]. Olsen et al. explained the fluttering of a double-clamped membrane by Karman vortex shredding and gave an easy-to-use formula to calculate the vibration frequency based on the Strouhal number [36]. Numerical simulations were also conducted on membrane flutter and vibration with bending and torsion under various vibration frequencies [34,38,39]. All the above approaches are worth learning from.

However, few studies fully investigated the effect of the narrow tunnel on the airflow and the electrostatics on the membrane movement. On one hand, the confined airflow (disturbed by the fluttering membrane) is completely different from the flow in the open space due to the difference in boundary conditions. On the other hand, in the real situation with the narrow tunnel, two polarized electrodes on both sides of the fluttering membrane will attract the membrane since the membrane and its nearest electrode will always carry charges of opposite polarity. Thus, as it oscillates, the membrane does not tend to stay in the middle but rather moves randomly to either electrode quickly. As the film reaches one electrode, the wind will blow away the membrane from the nearest electrode, creating rhythmic oscillation and smooth energy harvesting cycles. Therefore, the effect of confined flow and electrostatics should be considered in future theoretical and computational fluid dynamics studies on fluttering TENGs.

The detailed information of wind flutter-type sensors is summarized in Table 2, including wind speed range, wind direction, and features. The tested wind speed for the flutter-type sensors is from 0.5 to 32.0 m/s (a few studies without TENG tried much higher wind speed [32,34]). Similar to the conventional sensors with wind cups, the current trend is to correlate wind speed with electrical signal frequency, whose advantages were discussed in the previous subsection. However, the fluttering frequency has upper limits according to membrane material, structure, and natural frequency so the corresponding flow velocity that can be measured is limited. One way to increase the frequency is to change to the membrane with a higher upper limit for fluid speed, e.g., thin stainless steel foil; another way is to reduce the flow speed at the interested zones by expanding the tunnel cross-section along the flow direction [40,41] according to Bernoulli principle [42]. Thus, the measured frequency can represent higher wind speed, increasing the possible speed sensing range and expanding the application scenarios, e.g., airspeed monitoring for aircraft.

The flow direction can be measured by flutter- and flag-type sensors in two ways, similar to the traditional wind cup types. The first way is also using a wind vane to guide the device [43,44]. Another way is to use an array of direction-sensitive sensors [45] that have one (e.g., one end clamped types) or two (e.g., double-clamped types in Figure 5f [46]) favorable wind directions, a feature that is a drawback for energy harvesting, but a great benefit for sensing. Thus, by locating the sensors in the desired direction, the combined system can deliver the wind direction.

Compared to other types of sensors, flutter- and flag-type sensors have some unique advantages. First, their design is simpler and thus easier to manufacture. Second, they own minimum moving parts and don’t contain gears, making them small in size in general with great scalability, which could be critical for expanding their application scenarios in the microscopic world. Finally, with minimum moving parts and mainly contact and separation motion, their robustness is much better than the rotational and frictional-based sensors.

**Figure 6:**
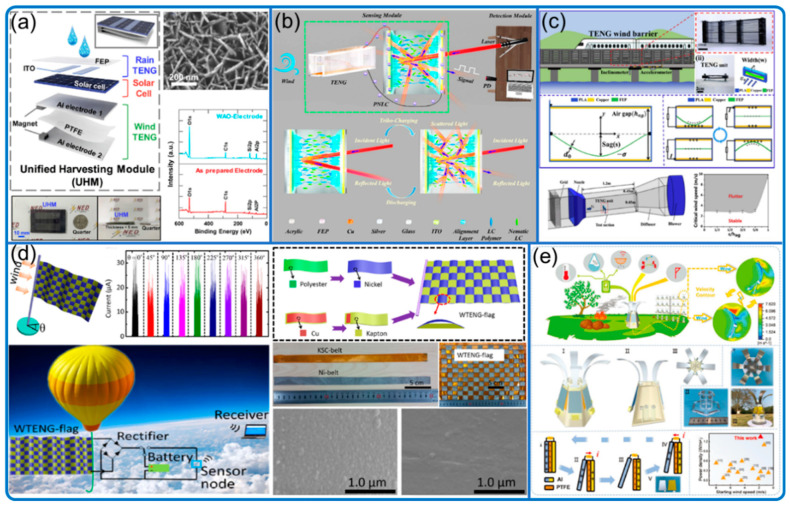
Flutter sensors’ special functions and features. (**a**) Weather monitoring system. Reproduced with permission. Copyright 2020, Elsevier [47]. (**b**) Mechanical−electrical−optical signal conversion system. Reproduced with permission. Copyright 2021, American Chemical Society [49]. (**c**) Sensors as wind barrier. Reproduced with permission. Copyright 2020, Elsevier [46]. (**d**) Woven flag-type system. Reproduced with permission. Copyright 2016, American Chemical Society [48]. (**e**) Multidirectional fire detection system. Reproduced with permission. Copyright 2021, Wiley Online Library [45].

Benefitting from the mentioned advantages, in addition to being the energy harvester and sensor, the flutter- and flag-type sensors have many other special functions and features as well (Figure 6). For example, Wang et al. (Figure 6c) constructed a flutter array system to work effectively as a wind barrier [46]. Roh et al. (Figure 6a) integrated the flutter sensor into a hybrid environmental energy harvesting and weather monitoring system [47]. Zhao et al. (Figure 6d) fabricated woven flag-type TENG in the size of 30 × 30 cm^2^ with triboelectric and conductive belts [48]. Furthermore, Wang et al. (Figure 6b) achieved wireless sensing using mechanical−electrical−optical signal conversion, where the airflow speed is correlated with the light signal that is received remotely by the optical detection module [49].

**Table 2:** Summary of flutter- and flag-type TENG sensors.

Reference	Wind Speed (m/s)	Wind Direction	Features
Yang et al. [31]	6.0 to 14.0	Yes, arrayed devices	The earliest flutter-type TENG
Zhao et al. [48]	3.0 to 32.0	N/A	Flag-type device with fabricated belts
Ravichandran et al. [40]	0.5 to 10	N/A	Venturi flutter-type; the interested zone wind speed is increased due to Bernoulli principle
Wang et al. [44]	2.0 to 7.5	Yes, circuit indicator with 4 directions	Flag-type with humidity resistance
Wang et al. [46]	4.0 to 11.0	N/A	Flutter-type TENGs as wind barrier
Zaw et al. [50]	1.5 to 2.7	Yes, wind direction sensitive	Flutter-type but ripped style TENG.
Roh et al. [47]	6.0 to 13.0	N/A	Weather monitoring system with rain and wind flutter TENG with solar panel
Li et al. [51]	1.6 to 14.0	N/A	Flutter-type TENG with carbon nano thorn arrays on the electrode
Liu et al. [41]	15.0 to 25.0	N/A	Flutter-type with expanded chamber
Xu et al. [43]	2.9 to 24.0	Yes	Flutter-type; photoelectric sensor for direction and wind blow toward the long side
Wang et al. [49]	2.5 to 10.0	N/A	Flutter-type; wireless sensing with mechanical−electrical−optical signal conversion
Zhang et al. [45]	1.8 to 4.3	Yes	Flow induced vibration; 6 wind directions by array; fire detection

**Figure 7:**
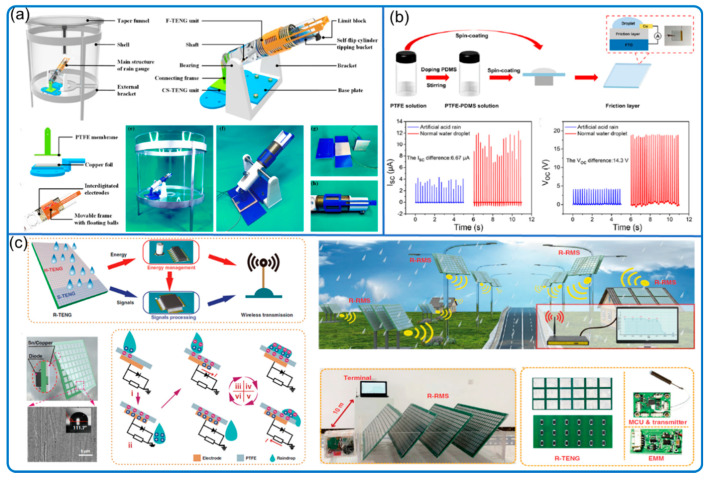
Rain monitoring TENG. (**a**) Tipping bucket rain gauge sensor. Reproduced with permission. Copyright 2022, Elsevier [53]. (**b**) Real-time acid rain sensor. Reproduced with permission. Copyright 2021, American Chemical Society [57]. (**c**) Raindrop-powered autonomous rainfall monitoring. Reproduced with permission. Copyright 2022, Springer [56].

(c) Rain monitoring:

Another meteorology sensing using TENG is rain monitoring. The traditional rain gauges mainly include siphoning, weighing, and tipping bucket types [52]. Similar to the traditional gauges, Hu et al. (Figure 7a) developed two rain bucket method mechanical TENGs with different resolutions to monitor the rain bucket motion change for real-time rainfall intensity monitoring from 0 to 288 mm/day with linear calibration curves [53].

In addition to the traditional rain gauge style sensor, the direct interaction between the raindrop and sensitive materials can be another possible way to sense the rain. In fact, the TENG field has extensive studies on solid–liquid interface contact electrification [54,55]. However, many of them focus on fundamental mechanisms and energy harvesting; few studies focus on rain monitoring. Until recently, Xu et al. (Figure 7c) designed autonomous rainfall monitoring and wireless transmission system completely driven by raindrop energy harvesting, paving the way for raindrop-powered wireless hygrometers [56]. The tested rain intensity is from 2.0 to 71.0 mm/min. When the rainfall is 71 mm/min, the rainfall data are automatically transmitted every 4 min within the range of 10 m. Another study (Figure 7b) takes advantage of the effect of hydrogen ion concentration on the material response and consequent TENG output to develop a pH sensor for acid rain [57].

### 3.2. Fluid Sensing in Pipes and Tunnels

**Figure 8:**
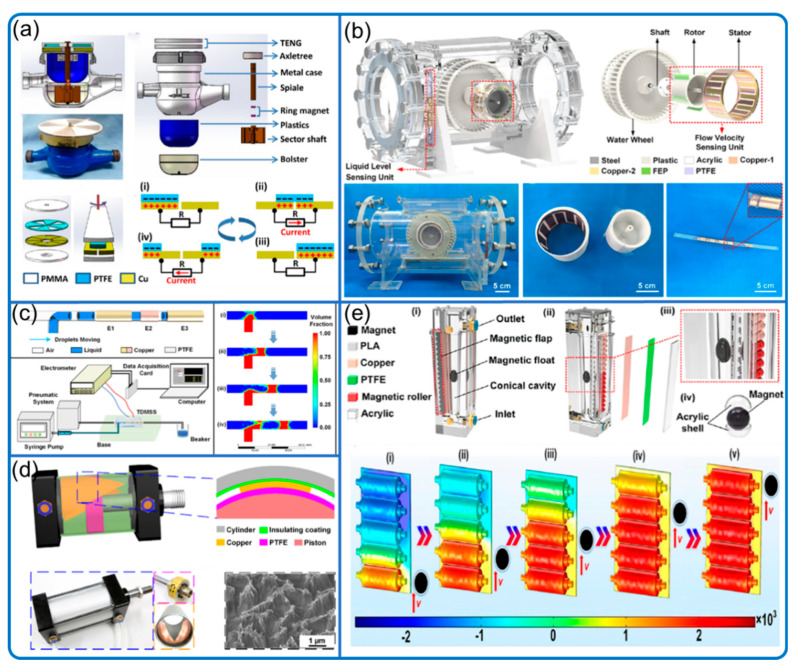
Fluid sensing in pipes and tunnels. (**a**) Anti-rust waterwheel-type system. Reproduced with permission. Copyright 2019, American Chemical Society [58]. (**b**) Non-full pipe fluidic flow and water level system. Reproduced with permission. Copyright 2022, American Chemical Society [59]. (**c**) Non-invasive droplet motion monitoring. Reproduced with permission. Copyright 2021, American Chemical Society [60]. (**d**) Pneumatic monitoring with varied-area electrodes. Reproduced with permission. Copyright 2017, American Chemical Society [63]. (**e**) Magnetic flap-type rotameter-like difunctional sensor. Reproduced with permission. Copyright 2020, American Chemical Society [66].

Pipes and tunnels are other places where fluid dynamics sensing is important and suitable. A network of self-powered sensors will be important for cost reduction and risk alarming in the industry of pipeline transportation. Thus, researchers were exploring TENGs for fluid sensing in pipes and tunnels with three main types (Figure 8):

(a) Waterwheel-type

Wang et al. (Figure 8a) developed a waterwheel-type flow rate sensor in pipes from 80 to 400 mL/s (i.e., 4.8 to 24.0 L/min) with an anti-rust feature [58]. Similarly, He et al. (Figure 8b) developed a non-full pipe flow sensor (220 mm in diameter and 105 mm in width) monitoring the flow rate from 94 to 264 L/min, with an error rate under 1.95% [59].

(b) Solid–liquid interface contact electrification

Another sensor in He et al. [59] is a liquid-level sensor applying solid–liquid interface contact electrification. Using the same principle, Song et al. (Figure 8c) developed a sensor for microfluidic droplet sensing with good stability and responsiveness [60]. It also has good sensitivity to the droplet size with the droplet length from 3.0 to 13.5 mm. Zhang et al. developed liquid in a U-tube TENG for air pressure sensing (from about 0.1 to 0.54 kPa), where air humidity did not affect performance [61]. Cui et al. also developed a liquid in solid tube-based TENG sensor monitoring air pressure from −2.5 to −0.5 kPa and 0.5 to 2.5 kPa [62].

(c) Cylinder-type

Fu et al. (Figure 8d) applied a cylinder moved through varied area electrodes by air pressure to detect air pressure and flow rate in the tunnel with the pressure from 0.04 to 0.12 MPa at a step of 0.02 MPa and flowrate from 50 to 250 L/min at a step of 50 L/min [63]. The way the authors varied the area of detection, which is also used in other fields [64,65], to generate tunable signals is interesting and very useful in signal manipulation. Wang et al. (Figure 8e) later developed a rotameter style TENG sensor for liquid level and pneumatic flow from 10 to 200 L/min with a flow resolution of 2 L/min with real-time non-digital flow rate visualization [66].

### 3.3. Remote Media Vibration and Object Sensing

**Figure 9:**
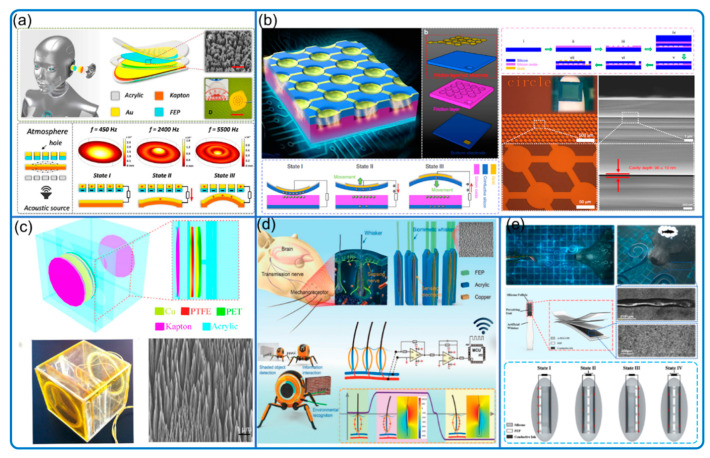
Remote media vibration sensor. (**a**) Auditory sensor. Reproduced with permission. Copyright 2018, AAAS [67]. (**b**) Micro triboelectric ultrasonic device. Reproduced with permission. Copyright 2020, American Chemical Society [69]. (**c**) Acoustic source locator in underwater environment. Reproduced with permission. Copyright 2015, Springer [70]. (**d**) Biomimetic hairy whiskers. Reproduced with permission. Copyright 2021, Wiley Online Library [71]. (**e**) Underwater bionic whisker sensor. Reproduced with permission. Copyright 2022, Elsevier [72].

Sound, a kind of acoustic wave, causes media vibration that can be detected by TENG sensors [67,68,69] (Figure 9). Arora et al. demonstrated an acoustic sensor (microphone) that is thin, flexible, and easily manufactured [68]. Its resonant frequency is approximately 275 Hz and the best acoustic sensitivity is −26.63 dB at 1000 Hz. Similarly, Guo et al. (Figure 9a) applied thin film TENG with holes and developed an auditory sensor for social robotics and hearing aids [67]. It has a broadband response from 100 to 5000 Hz and ultrahigh signal sensitivity (110 mV/dB). Chen et al. (Figure 9b) later claimed to have developed the smallest MEMS (micro-electro-mechanical systems) TENG device with micro-array in 50 µm-sized diaphragms, achieving underwater acoustic communication within 30 mm. Its signal-to-noise ratio (SNR) is 20.54 dB and its resonant frequency is as high as 1.17 MHz [69].

The media vibration caused by objects, not only due to sound waves but also by surrounding water and air movement, can be also detected by TENG sensors, with appropriate algorithms [70,71,72]. Yu et al. (Figure 9c) developed an organic film TENG as an underwater acoustic source locator at low frequencies around 100 Hz [70]. It can detect up to 1.0 m with an error of about 0.2 m and the highest sensitivity –146 dB. Wang et al. (Figure 9e) developed a seal whisker-like, film-type sensor for underwater vortex perception and demonstrated its target tracking capabilities, with an SNR of about 19 dB without filtering [72]. As demonstrated, TENGs as vortex sensors should be further investigated and could be very useful for the fundamental study of fluid dynamics. In An et al. (Figure 9d), a bendable biomimetic whisker mechanoreceptor (film-type TENG) is designed for robotic tactile sensing [71]. The sensor has a very high SNR with a minimum exciting force of 1.129 μN thanks to the simple and functional design, making it also suitable to detect natural flow vibration and object-induced air movement. Furthermore, the sensor can be scaled down to a very small size and integrated within the airflow boundary layer, whose airflow situation can be sensed with minimal device impact.

### 3.4. Water Wave Motion

**Figure 10:**
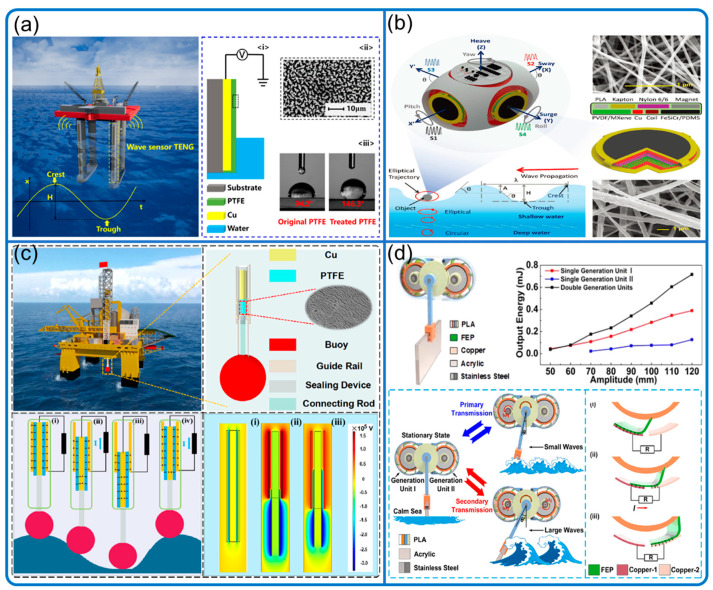
Sea wave motion sensing. (**a**) Highly sensitive wave sensor. Reproduced with permission. Copyright 2019, Elsevier [73]. (**b**) Self-powered arbitrary wave motion sensing system. Reproduced with permission. Copyright 2022, Wiley Online Library [78]. (**c**) Ocean surface water wave sensor. Reproduced with permission. Copyright 2020, American Chemical Society [75]. (**d**) Ocean wave sensor with graded energy harvesting. Reproduced with permission. Copyright 2021, American Chemical Society [76].

We roughly classify the TENG sea wave sensors into floating and fixed types (Figure 10). The floating types generally have more flexibility for setup and wider application scenarios than the fixed ones. The sensing mechanism is mainly solid–solid contact electrification, while a few studies applied liquid–solid contact electrification.

The wave parameters sensed here include wave height, magnitude, fluctuation, and other local parameters. For example, wave height can be sensed by the fixed liquid–solid plate TENG (Figure 10a) [73], the coral-like four-way TENG [74], and the buoy-driven TENG (Figure 10c) [75]; wave magnitude can be monitored in two grades by the TENG with cam gear (Figure 10d) [76]; wave surface fluctuation can be monitored by the floating jellyfish-like TENG with various working scenarios [77]. In addition to large-scale sensing, Bhatta et al. (Figure 10b) developed a powerful floating TENG-EMG hybrid wave motion sensor, offering various comprehensive wave-related local parameters, such as the wave speed, acceleration, frequency, direction, tilting angles, and wavelength with good accuracy [78].

### 3.5. Structural Vibration Due to Moving Fluid

Flow-induced structural vibration is an area of interest and concern in both academia and industry. Especially in industry, it is a big problem for the safety of buildings, bridges, grid lines, and fast-moving vehicles in the air and water, but also an opportunity for energy harvesting, for example, the bladeless vibration energy harvester [79,80].

**Figure 11:**
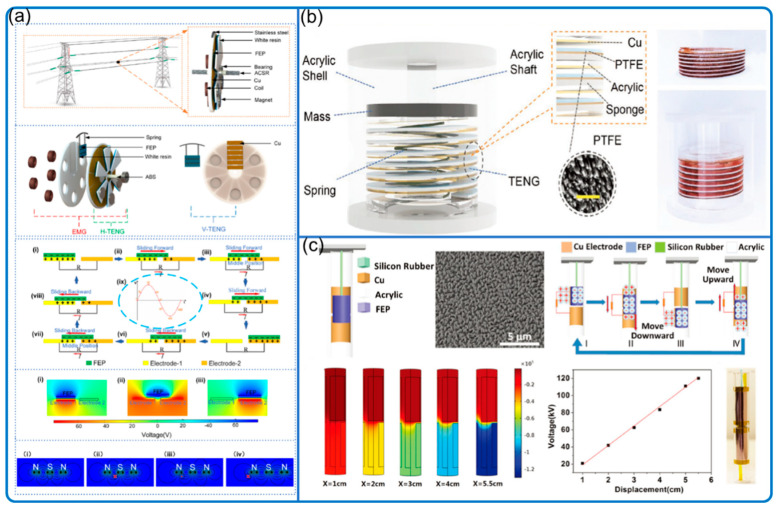
Structure vibration due to moving fluid. (**a**) Transmission lines galloping sensor. Reproduced with permission. Copyright 2022, Elsevier [81]. (**b**) Broadband aeolian vibration online monitoring of transmission lines. Reproduced with permission. Copyright 2022, Wiley Online Library [82]. (**c**) Bridge dynamic displacement monitoring system. Reproduced with permission. Copyright 2017, Wiley Online Library [84].

Due to their cyclic nature, these vibrations, usually rhythmic, can be also monitored by TENG sensors (Figure 11). For example, power line galloping (self-excited vibration phenomenon with a low frequency) and aeolian (wind excitation phenomenon with high frequency) sensors have been developed by Gao et al. and Wu et al., respectively [81,82]. Gao et al. (Figure 11a) developed a TENG-EMG hybrid generator (pendulum-type) with grid line galloping energy harvesting and monitoring [81]. The horizontal, vertical, and elliptical galloping modes were physically simulated and tested by a scale reduction model, and the amplitude and frequency of the three modes were monitored over a range of 5.0 to 16.8 cm (with linear calibration curves) and 0.7 to 2.2 Hz, respectively. The broadband aeolian vibration sensing and its effective energy harvesting of transmission lines were achieved by a spring-mass type TENG (Figure 11b) in Wu et al. [82]. The sensed amplitude is from about 0.5 to 6.0 mm, with a broad frequency response region of 5–50 Hz. Unfortunately, the vibrations in both of the studies were not induced by airflow under a real aerodynamic situation, which is needed to further validate the senor performance in practical applications.

Bridge vibration can be also induced by moving fluid: either by (1) forced resonance caused by vortex shredding, or (2) aeroelastic self-excitation or “negative damping” flutter effect. For example, the dramatic collapse of the Tacoma Narrows Bridge was related to an aerodynamically induced vibration, a combination of forced and self-excited vibrations, where the critical cause is still under debate [83]. To monitor the bridge dynamic displacement (1.0 to 5.5 cm) and vibration acceleration (13.7 to 49.0 ms^−2^), Yu et al. (Figure 11c) designed a dynamic displacement monitoring system (spring-mass in cylinder style) within a low-frequency working range (<5–10 Hz) [84].

## 4. TENG for Fluid Dynamics Sensing: Future

The above section has summarized many applications of TENG in fluid dynamics sensing. However, to reveal the full potential of TENG for fluid dynamics sensing, we need to take advantage of TENG, and ultimately fill the gaps and meet the needs of industry and academia. In academia, alternative methods are needed to measure the in situ local fluid situations, e.g., vortex, boundary layer, and turbulence transition. The most reliable method to observe fluid dynamics is optical techniques, such as particle image velocimetry (PIV) and Schlieren imaging. However, these techniques require a sophisticated setup and critical experimental environment in the laboratory, making them not suitable for multi-scale field measurements, which play very important roles in the validation and application of fundamental theories.

In industry, especially aerospace, non-invasive sensors are in large demand, for flight safety, flight data acquisition, and aircraft design optimization by monitoring the local in situ fluid situation in real flight. The critical parameters include aircraft airspeed, friction drag force, vortex and boundary layer separation near the wing surface, and wing vibration. However, most of the traditional sensors need power with cables that will increase the weight. In addition, since most third-party sensors are not pre-designed in the body, the power supply and cables of the sensors will expose to the extreme atmosphere, creating a difficult scenario for high-speed aircraft because the fast-moving air may damage the cables, which also in turn disturb the airflow field and thus the flight condition and safety. As for light-weighted unmanned aerial vehicles (UAV), the increased weight from the whole sensor system could be another critical problem for efficient flight. Thus, the industry craves suitable sensors with the features of lightweight, plug-and-play, in situ, and battery-less, so that significant progress can be made in the aerodynamics theory development, aircraft design, and safety monitoring.

**Figure 12:**
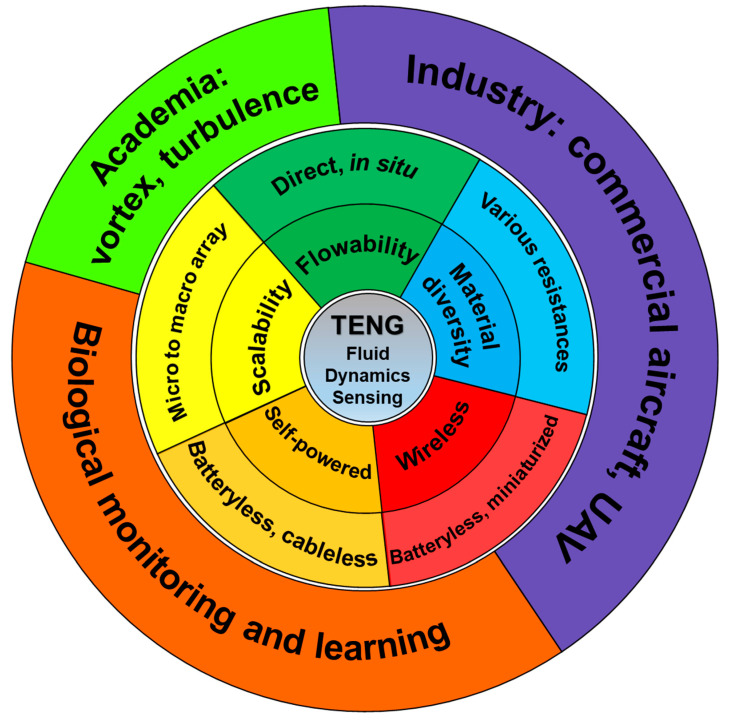
Schematics of advantages and future applications of TENGs for fluid dynamics sensing.

Fortunately, TENG sensors have the potential to fulfill some of the above needs in fluid dynamics sensing thanks to the following advantages: self-powered sensing, wireless signal transfer, scalability, material diversity, and flowability (Figure 12). As mentioned in Section 1, TENGs can actively generate electrical signals and have the potential to generate enough power to save and even transmit signals by the TENG alone, making the sensor setup and maintenance much easier than the conventional ones. Tao et al. designed a hierarchical honeycomb-structured structure for morphing airplane energy harvest, claimed as the first TENG used for aircraft, showing the feasibility of energy harvesting and self-powered sensing in the aerospace industry [85].

Furthermore, with the feasibility exploration and approach development of wireless transmission of TENG signal in air and water [11,12,86,87], even if the TENG is not powerful enough to run the whole system, the sensing signal can be also possibly received without any power supply and the need of signal pre-amplification. Wang et al. summarized TENG-based self-powered wireless communication into four major types of technological routes: tribo-induced electromagnetic wave generation, tribo-induced light propagation tuning, triboelectrification-induced electroluminescence, and tribo-assisted spectrometry, collectively known as tribophotonics [12].

In addition to signal generation and transferring, the adaptive design and easy manufacturing, thanks to its simple working mechanism and various working modes, endow TENGs with strong scalability, meaning that the complexity and difficulty of manufacturing are not heavily affected by the size of the devices. Many design techniques are highly scalable by avoiding the use of unscalable parts, such as traditional joints, screws, nuts, and gears. For example, compliant mechanism design can reduce parts, joints, and production processes, making it advantageous in the fabrication of micro-mechanisms [88]; origami and kirigami design [89] can easily transform a device into a 3D structure with the desired function from a 2D design, which is convenient for industrial-level and TENG friendly precision manufacturing techniques such as laser cutting, pre-stretching, printed circuit board (PCB), and microelectromechanical systems (MEMS).

Consequently, the scalability gives TENG sensors better chances to form an array [69] and get applied to the microenvironment. For example, a rationally designed TENG can locate within the flow boundary layer and provide valuable local information for flow fundamental research. In addition, an array of such sensors can even deliver more comprehensive information in high spatial and temporal resolution [90]. In addition, TENG sensors with good scalability (better if with wireless communication) can be used for biological monitoring, data acquisition, and learning, where we can learn deeper and better from nature, especially its fluid-related phenomenon [91,92], e.g., flying of birds and swimming of fishes, allowing us to have innovations and breakthroughs in the engineering development for machines, vehicles, and robotics [93,94]. Of course, this influence has already penetrated deep into the TENG field [95,96,97]. For an instance, the study of seal whisker (vibrissa) suppressing vortex-induced vibrations provides us with a possible solution to increase the SNR (signal-to-noise ratio) for a TENG array system of object-induced vortex detection [98]. However, the investigation tools and approaches to study nature are limited. Tiny, lightweight, and scalable TENG sensor arrays will make a difference in the in situ fluid dynamics monitoring of living beings.

The material diversity that offers a wide selection of materials is another unique feature of TENG sensors, especially for fluid dynamics sensing. Fluids may contain a variety of physical and chemical phenomena that make most sensors difficult to deploy and function. The situation is much better for TENG sensors, as almost any two materials can generate a high or low triboelectric signal so that we can choose the right ones from the large material library to suit specific, possibly extreme, and multi-physics environments (e.g., high temperature, pressure, electrical noise, bending, and friction environments) with better physicochemical performance such as high chemical resistance, hydrophobic/hydrophilic, temperature resistance (e.g., titanium–tantalum alloy), fatigue resistance, and shape memory (e.g., nitinol alloy), and light-weighted but high strength (e.g., carbon fiber). For example, similar to the flutter-type speed sensor, a thin metal foil, which can flow along with air, can be used as the fluttering material to sense high wind speed in high-temperature situations (e.g., airspeed sensing for aircraft) instead of polymer films, which are fragile with low-temperature resistance.

Another important property of TENGs for fluid dynamics sensing is flowability, which means that the TENG can conform to and move along with the moving fluid, making it very unique in the fluid sensing field. Most of the traditional sensors are fixed rigidly and cannot move with the fluid. A moving along sensor can give detailed and valuable in situ information on the fluid local status including vortex [99,100], boundary layer separation, and turbulence, which are some of the most difficult subjects for fundamental fluid research and critical for the field of fasting moving vehicles such as aircraft and submarine. As for those traditional fixed sensors for fluid dynamics, only the related but indirect parameters can be measured to calculate the interested parameters with many assumptions and simplifications. For example, the MEMS skin sensors for aircraft measure the fluid-related parameters, such as pressure and temperature, and indicate the flow turbulence and stall status indirectly through calculation with mathematical algorithms [101,102].

In this section, we have summarized the unique advantages of TENG in fluid dynamics sensing (including self-powered, wireless communication, flowability, a wide selection of material, and scalability) and their corresponding potential new applications that differ from the previous ones (Section 3). Hopefully, the new perspective can help TENG reach its full potential by providing brand-new and powerful tools and data for academia and industry in the new era of IoT and AI and offering more chances for TENG to constantly develop and show its ability.

## 5. Challenges and Possible Solutions

The three most important features and also the main challenges for qualified sensors, including TENG sensors, are sensitivity, selectivity, and stability. In this section, we discuss several challenges that TENGs are facing to consolidate their positions in the existing areas and reach their full potential and capabilities in the key new fields of fluid dynamics sensing and provide helpful tools and possible solutions (Figure 1).

Researchers need to master the fundamental knowledge and understanding of fluid dynamics, fluid mechanics, and aerodynamics for aircraft, so that (a) we know where to target and measure, and what to enhance and avoid; (b) we can choose the right TENG material and design with proper structure and casing to suit the fluid situation; (c) we can choose the right supporting calibration equipment to observe, calibrate, and validate the TENG sensors and eventually enhance their performance, according to the theories.

Here are some basic and, most importantly, easy-to-use principles and equations, especially with explicit solutions, to explain and analyze fluid dynamics:

(a) For inviscid flow, if we assume the fluid motion is governed by pressure and gravity forces only, Newton’s second law applied to a fluid particle is in the form:

(Net pressure force on partaicle)+(net gravity force on particle)=(partice mass)×(particle accelaration)

As a result of the interplay among pressure, gravity, and acceleration, fluid mechanics has numerous useful applications. Furthermore, when applying Newton’s second law on a fluid particle along the streamline, we obtained the celebrated Bernoulli equation.

(b) Bernoulli equation: a very powerful tool in fluid mechanics

$$p+\frac{1}{2}\rho V^{2}+\rho gz=\mathrm{constant}\;\mathrm{along}\;\mathrm{streamline}$$

where *p* is the fluid pressure, ρ is the fluid density, *V* is the flow velocity, *g* is the gravitational constant, and *z* is the elevation term. The equation basically means the sum of the pressure term, velocity term, and elevation term is constant along a streamline. Note that to use the equation correctly we must always remember the basic assumptions used to derive it: (1) negligible viscous effects, (2) steady flow, (3) incompressible flow, and (4) the equation is applicable along a streamline. This equation can be used conveniently together with the Coanda effect [103] which is the tendency of a fluid jet to stay attached to a curved surface, where the fluid will speed up and, according to the Bernoulli equation, has low pressure.

(c) Navier–Stokes equations: governing differential equations for incompressible Newtonian fluids

$$\rho \left(\frac{\partial V}{\partial t}+V\cdot \nabla V\right)=-\nabla P+\rho g+\mu \nabla^{2}V$$

Above is the form compactly expressed in vector notation. Detailed differential equations can be found in Munson et al. [42]. The Navier–Stokes equations are essential to all fluid mechanics. The equations’ analytical solution has not been found yet; however, the numerical solution can be used in computational fluid dynamics (CFD), which is another powerful tool in understanding and explaining the fluid dynamics phenomena from a different perspective.

(d) Reynolds’ number: generally, of importance in all types of fluid dynamics problems

$$R_{e}=\frac{\rho Vl}{\mu }$$

where *l* is the characteristic length, μ is the fluid viscosity. This dimensionless number should match between simulations and experiments for comparable and accurate data. In addition, it could be used as a criterion to distinguish between laminar and turbulent flow. Note that the criteria are different according to the characteristic length and the scenarios, such as flow in the pipe and flow around the object.

(e) Strouhal number: a dimensionless number describing unsteady flow with a characteristic frequency of oscillation

$$S_{t}=\frac{\omega l}{V}$$

where ω is the frequency of vortex shedding, *l* is the characteristic length (for example, hydraulic diameter or the airfoil thickness), and V is the flow velocity. This number can be used to investigate the flow-induced vibration such as bridges and grid lines vibration in the wind, and vortex shredding in the water.

1. The testing, calibration, and validation instruments are needed for the proper design of TENG fluid dynamics sensors. This is also a challenge because the equipment is either expensive and difficult to handle or limited. To avoid discrepancy caused by the noise from the testing environment, we need well-controlled water and wind tunnels, (e.g., high speed or low turbulence wind tunnel) and optical observation systems, e.g., PIV (particle image velocimetry) flow velocity distribution measurement and Schlieren imaging systems. Inaccurate and loose testing apparatus may generate inaccurate calibration curves and deliver biased results during sensor design and testing phases. In addition, signal calibration and subsequent readjustment algorithms are also critical for pushing the instrument response to the desired parameters [64,65].

2. To take advantage of the scalability of TENG sensors, we must keep in mind scalable and parametric design, and the corresponding manufacturing techniques. For example, for the fluttering sensor, the system size is not as critical when used in an ordinary environment; however, when used for the flow boundary layer sensing, the system needs to be as thin and small as possible to locate within the boundary layer and minimize the system disturbance to the flow. Thus, if scalability is kept in mind at the beginning of design, the sensor system can be easily transferred from a normal to a specific environment. Otherwise, any unscalable techniques (e.g., low-resolution techniques and parts) during the manufacturing process when changing scales will prevent the device from being scalable.

3. The core function of sensors, including TENG sensors, is signal selectivity, which is distinguishing different signals by responding selectively to the interested signals and filtering out noise signals. In other words, the system must be either very selective or the sensing process can be strictly controlled so that uninterested signals will not interfere with measurements. Therefore, sensor design for fluid dynamics must be very careful with assumptions and algorithms, since many indirect sensors rely on a stable environment and assume constant environmental parameters, such as fluid density, temperature, and pressure, which could be invalid during realistic measurements. This is also why in situ sensors with direct measurement (also the focus of TENG sensors) are always preferred when possible, with fewer assumptions to make and fewer variables to control. Furthermore, field measurements with realistic and multi-variables situations are required to ultimately validate the sensors [65].

4. Even if the sensors are well designed and calibrated, the multiphysics environment (water, high temperature, electrical field, and plasma shield) often found in fluids can still reduce the performance and lifespan of devices and disturb the signal generation and transmission. Thus, the solution is to find and apply the optimal design and materials, which are the unique advantages of TENGs, to cope with high bending, chemical, and humidity conditions, and to generate signals with a generally high SNR (signal-to-noise ratio).
